# The trAPP-study: cost-effectiveness of an unsupervised e-health supported neuromuscular training program for the treatment of acute ankle sprains in general practice: design of a randomized controlled trial

**DOI:** 10.1186/s12891-015-0539-9

**Published:** 2015-04-09

**Authors:** Adinda KE Mailuhu, Evert ALM Verhagen, John M van Ochten, Patrick JE Bindels, Sita MA Bierma-Zeinstra, Marienke van Middelkoop

**Affiliations:** Department of General Practice, Erasmus MC, University Medical Centre, Rotterdam, PO Box 2040, 3000 CA Rotterdam, The Netherlands; Department of Health Sciences and EMGO Institute for Health and Care Research, VU University Medical Center, Van der Boechorststraat 7, 1081 BT Amsterdam, The Netherlands

**Keywords:** Ankle sprains, Treatment, Neuromuscular training

## Abstract

**Background:**

Ankle sprains are one of the most frequent injuries of the musculoskeletal system, with yearly around 680.000 new sprains in the Netherlands. Of these, about 130.000 people will visit the general practitioner (GP) each year. In addition, patients have an increased risk of a recurrent ankle sprain and about a third report at least one re-sprain. No optimal treatment strategy has proven to be effective in general practice, however promising results were achieved in a preventive trial among athletes. Therefore, the objective is to examine the (cost)-effectiveness of an unsupervised e-health supported neuromuscular training program in combination with usual care in general practice compared to usual care alone in patients with acute ankle sprains in general practice.

**Method/Design:**

This study is a multi-center, open-label randomized controlled trial, with a one-year follow-up. Patients with an acute lateral ankle sprain, aged between 14 and 65 years and visiting the GP within three weeks of injury are eligible for inclusion. Patients will be randomized in two study groups. The intervention group will receive, in addition to usual care, a standardized eight-week neuromuscular training program guided by an App. The control group will receive usual care in general practice alone. The primary outcome of this study is the total number of ankle sprain recurrences reported during one year follow-up. Secondary outcomes are subjective recovery after one year follow-up, pain at rest and during activity, function, return to sport, cost-effectiveness and compliance of the intervention. Measurements will take place monthly for the study period of 12 months after baseline measurement.

**Discussion:**

For general practitioners the treatment of acute ankle sprains is a challenge. A neuromuscular training program that has proven to be effective for athletes might be a direct treatment tool for acute ankle sprains in general practice. Positive results of this randomized controlled trial can lead to changes in practice guidelines for general practitioners. In addition, since this training program is e-health supported, positive results can also lead to a novel way of injury prevention.

**Trial registration:**

Dutch Trial Registration: NTR4765.

## Background

Ankle injuries are one of the most frequent injuries of the musculoskeletal system, with annually around 680.000 new sprains in the Netherlands [1]. This is about 15 percent of the total number of sport injuries. About 43% of the people who sustain an ankle injury will visit the general practitioner (GP) or, on their own initiative, a physiotherapist or emergency department [1]. The GP treats about 130.000 ankle injuries each year. In addition, physiotherapists see about 180.000 ankle injuries per year. Of all ankle injuries occurring annually, about 70% are ankle sprains [1]. The incidence of ankle injuries is approximately 18 new injuries per year per general practice, with the highest incidence seen in patients aged between 15 and 24 [2].

The occurrence of acute lateral ankle sprains can have large societal impact since the clinical course of these injuries is poor. About one third of the patients report residual complaints after treatment, such as re-sprains, pain, loss of function or a feeling of instability [3]. In a recent observational study among patients who visited their GP 6–12 months earlier for a lateral ankle sprain, 47.5% of the patients experienced persistent complaints and only 17.5% regarded themselves completely recovered [4]. In addition to persistent complaints, patients have an increased risk of a recurrent sprain. Up to 34% of the patients report at least one re-sprain within a 3-year period after their initial sprain [3].

The occurrence of an ankle sprain does not only have a physical impact for patients but also has a reasonable impact on society. A Dutch study on ankle injuries in athletes showed that the mean total costs of one ankle sprain are approximately €360 [5]. The annual costs of ankle sprains in the Netherlands are hereby estimated at €187 million, of which 80% is due to productivity loss [5,6]. It is estimated that a total of €35 million can be saved per annum, by the use of an effective intervention program [6].

Almost 50% of all ankle injuries, that require medical treatment are initially seen by the GP. Thus, primary care plays an important role in the treatment of acute ankle sprains [1]. The guideline of the Dutch College of General Practitioners summarizes the evidence on the potential treatments for acute ankle sprains [7]. A frequent applied treatment is RICE (Rest, Ice, Compression, Elevation), but there is no strong evidence that RICE is an effective treatment option. Functional treatment with brace or tape is also advised for ligament ruptures, but again it is unclear from the literature which functional treatment is most effective [8,9]. According to the guideline for general practitioners, exercise therapy is recommended for patients with work activities or sport participation with a high risk on a recurrent ankle sprain. However, a systematic review of van Rijn et al. [10] showed that there is only very limited evidence for the effectiveness of supervised exercise therapy compared to usual care in patients with acute ankle sprains. Overall, the clinical guideline for general practitioners is not consistent and includes treatment strategies that have limited evidence. Since general practitioners play an important role in the treatment of these patients and in order to reduce persistent complaints and the risk of having a recurrent ankle sprain, an effective and pragmatic intervention in general practice is necessary.

Promising results were achieved in a preventive trial conducted among athletes in the Netherlands [11]. A non-supervised neuromuscular training program was effective in the prevention of re-sprains; the intervention program was associated with a 35% reduction in risk of recurrence, in both initially medically and non-medically treated patients [11]. A relative risk reduction of 0.45 (95% 0.28-0.72) was even found in the initially non-medically treated patients.

The use of this neuromuscular training program for the prevention of a recurrent ankle sprain is recommended by a multidisciplinary clinical practice guideline, developed with the aim to prevent further health impairment of patients with acute lateral ankle injuries and meant for all care providers who are involved in the treatment and guidance of patients with ankle injuries [12]. Additional support for the use of the neuromuscular training program, comes from a recent systematic review, which assessed and summarized the economic evidence regarding diagnostic test, treatment and prevention for lateral ankle sprains [13].

Especially because of the relatively young target patient population, e-health and self-management might be an ideal way to reach this population and to guide them in their treatment. Based on the effective neuromuscular training program of Hupperets et al. [11,14], the ‘Versterk je Enkel’ (‘Strengthen your Ankle’) App has been developed in cooperation with Veiligheid.nl (Consumer Safety Institute), a Dutch non-profit organization which focuses on the incidence and prevention of injuries (due to accidents or violence), including sport injuries. The use of e-health, m-health and self-management programs are highly promoted in today’s health care and might potentially play a vital role to increase the program compliance of patients. If indeed, the ‘Versterk je Enkel’ app is effective in reducing the number of re-sprains in patients, a very relevant and easy to implement new intervention for patients with acute ankle sprains will become available for primary care.

### Objectives

The primary objective of this study is:

To examine the effectiveness of an unsupervised neuromuscular training program in combination with usual care in general practice compared to usual care alone in patients with acute lateral ankle sprains in general practice in terms of the number of re-sprains reported.

The secondary objectives are:

1. To examine the effectiveness of an unsupervised neuromuscular training program in combination with usual care in general practice compared to usual care alone in patients with acute lateral ankle sprains in general practice in terms of pain, function and recovery.
2. To examine the cost-effectiveness of an unsupervised neuromuscular training program in combination with usual care in general practice compared to usual care alone in patients with acute lateral ankle sprains.

## Methods/Design

### Study design

**Figure 1:**
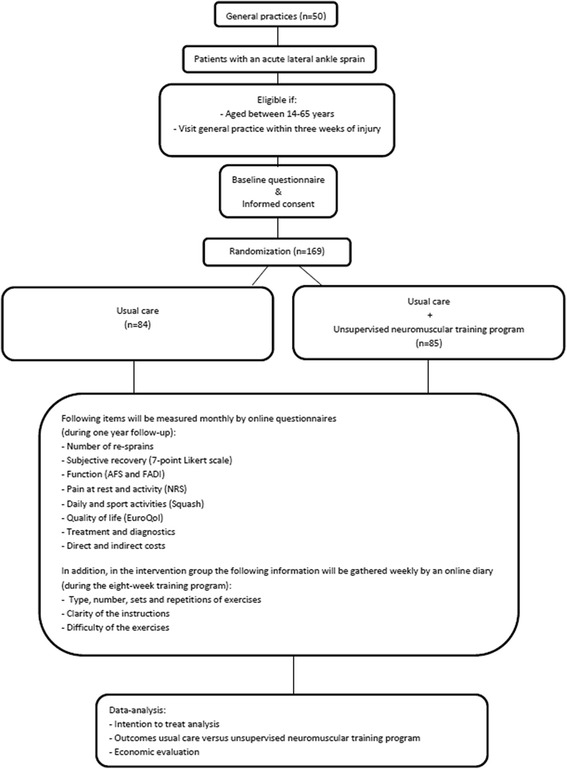
**TrAPP-study flow chart.**

The trAPP-study is a multi-center, open-label randomized controlled trial with a one-year follow-up. The study design and flow of participants are shown in Figure 1.

The study is funded by the Netherlands Organization for Health Research and Development (ZonMW). The study design, procedures and informed consent procedure are in compliance with the Declaration of Helsinki, 7th version, October 2013 [15]. The Medical Ethics Committee (number 2014–250) of the Erasmus University Medical Center, The Netherlands approved the study. The trial is registered in the Netherlands Trial Registry (NTR4765).

### Patient selection

Patients will be recruited by general practitioners located in the South West of the Netherlands. Patients with an acute lateral ankle sprain, aged between 14 and 65 years, who present themselves to a general practitioner within three weeks after an injury, are eligible for inclusion. Responders are excluded if they have a history of an injury of the same ankle during the previous year, they have had a fracture of the same ankle and if they have no understanding of Dutch language.

### Sample size

The sample size calculation is based upon the primary outcome measure ankle sprain recurrences. A previous randomized controlled trial found a difference of 64%, 53% and 25% respectively on the incidence of injuries per 1000 hours of sports for self-reported re-sprains, re-sprains that led to time loss and lead to costs [11]. A difference of 19% in the incidence of recurrent ankle sprains between the intervention and control group after a follow-up period of one year is considered to be clinically relevant. It is estimated that in 33% of the patients in the usual care group a re-sprain will occur during the 1-year follow-up period [11,16]. To detect the intended difference of 19% in the incidence of acute ankle sprain recurrences, with a power of 80% and an alpha of 0.05 (two-sided testing), a total of 77 patients per study group are needed. Taking a loss to follow-up of 10% into account, a total of 169 patients will be needed to include in the trial.

### Recruitment of study population

For our study we will recruit patients from general practices. Following the Dutch guideline ‘Ligament injuries ankle’ a total of 8 patients per 1000 patients are expected to be seen in general practice every year. On average, in a general practice approximately 18 new patients per year will be seen. It is estimated that approximately 30% of these patients in general practice are willing to participate in a trial. Therefore, a total of 31 participating general practices are needed to include a total of 169 patients within a year time. Taking into account lost to follow up and a lower participate rate, we will aim for 50 participating GPs.

Participating GPs will inform eligible patients about the study and ask them whether they are interested in participating in the study. All patients will receive an information leaflet with general information about ankle sprains, following the Dutch guideline. If patients are interested in participation in the study, they will fill in a reply card with contact details, together with the general practitioner or assistant, and send it to the research team. Subsequently, the research team will contact the interested patients, inform them about the study and check the inclusion criteria. If the patient fulfills the inclusion criteria and is willing to participate, additional information on the study together with an informed consent form, will be sent to the patient with a self-addressed envelope. He or she will be asked to sign the informed consent and send it to the research team. A baseline questionnaire will subsequently be send to the patients by email. All patients are consequently randomized by a researcher into one of the two groups, the intervention or control group.

### Randomisation procedure

The randomisation sequence is determined by an independent researcher from the department, with the use of a computer generated randomisation list. This list contains random blocks of 2,4 and 6. This sequence is secret for all involved researchers of the study.

### Interventions

Subjects allocated to the control group will only receive the leaflet with general information about ankle sprains, following the Dutch guideline and will receive usual care. The usual care provided by the general practitioner consists of rest, ice, compression and elevation (RICE). Patients are advised to resume daily activities as much as the pain allows. In case these treatments are not effective, the guideline advices to consider exercise therapy.

**Figure 2:**
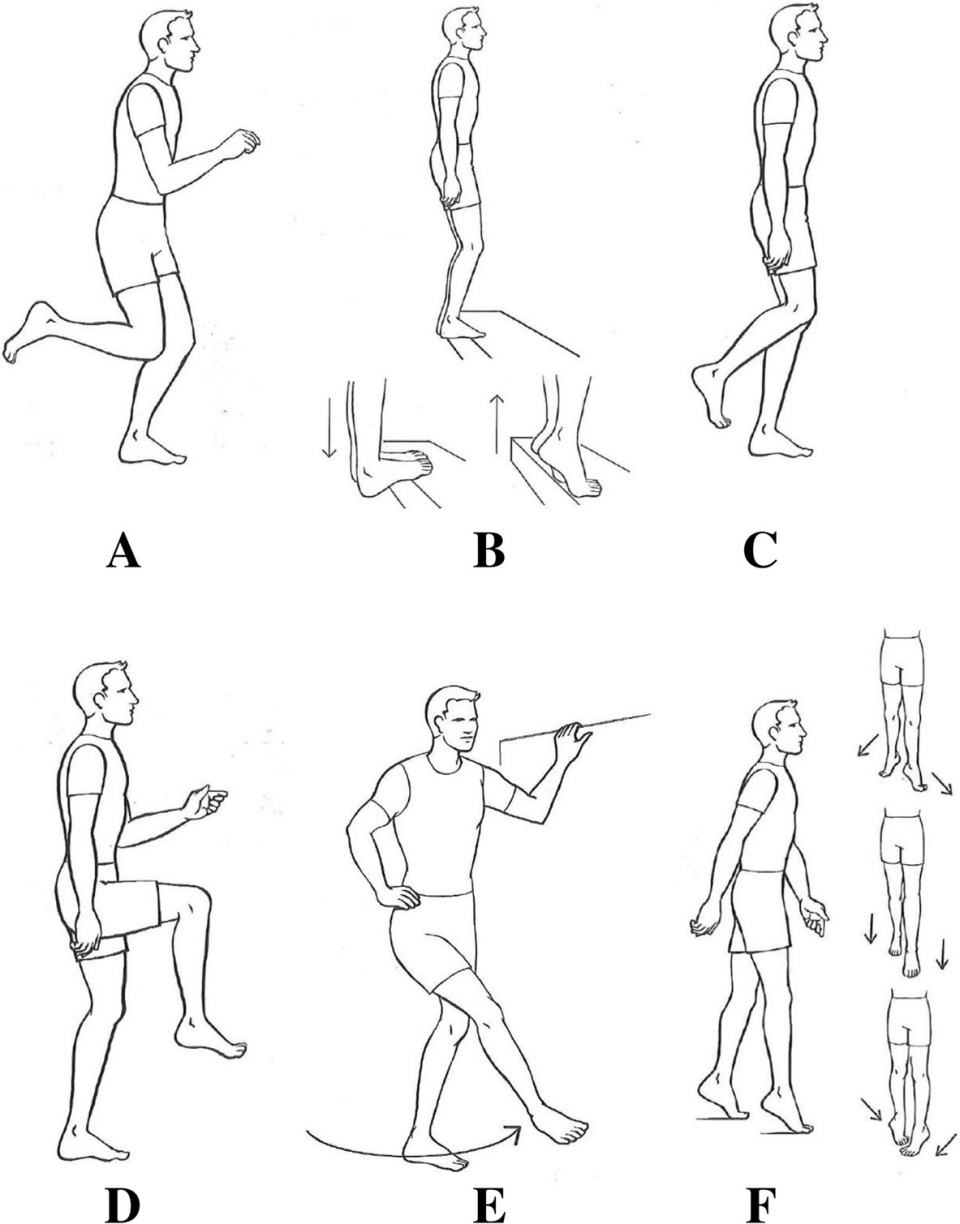
**Basic exercises of the neuromuscular training program** **[** 30 **]** **. A**. One-legged knee flexion. **B**. Toe stand. **C**. One-legged stance. **D**. Runner’s pose. **E**. Crossed leg-sway. **F**. Toe walk.

Subjects allocated to the intervention group receive a standardized neuromuscular training program, in addition to usual care. This neuromuscular training program has been proven to be effective for secondary prevention of ankle sprains within athletes [11]. The total duration of the intervention program is eight weeks. The frequency of exercising is consistent throughout the full eight weeks: the participants of the intervention group will perform three training sessions per week. Every training session consists of six different basic exercises (Figure 2). After several sessions, the exercises become more difficult, as the participant has to perform the exercises in different conditions (with eyes open or shut, with or without handhold, on even or uneven surface) [17].

All participants allocated to the intervention group will receive an e-mail with a hyperlink, referring to the application ‘Versterk je Enkel’ (free available for Apple and Android) [14]. In addition, they receive a flyer with general written and visual information and instructions on the exercises. The app is developed in collaboration with VeiligheidNL [14] and the content is based on the effective neuromuscular training program in a group of healthy athletes, studied by Hupperets et al. [11].

The app contains written and visual instructions of the basic exercises of the training program. In addition, the app includes general information about the use of brace or tape during sport participation. The participants are able to keep up with their exercises, using a personal scheme, integrated in the app. This personal scheme reminds the participants of the performed exercises and the following exercises, throughout the eight weeks of the training program.

All participants will train individually, without supervision of a coach or medical practitioner.

### Use of co-intervention

As part of usual care in general practice, participating general practitioners are free to apply a tape or brace, in both the treatment and control group. The use of co-interventions will be monitored during follow-up by monthly questionnaires.

### Study outcomes

The primary outcome of the trAPP-study is the total number of re-sprains reported after 1-year follow-up. A re-sprain is defined as an ankle sprain occurring as a result of sports participation or other daily activities and which cause one or more of the following [18]:

1. The subject has to stop the sports activity; and/or
2. Cannot (fully) participate in the next planned sports activity; and/or
3. Cannot go to work/school the next day; and/or
4. Needs medical attention (ranging from onsite care by e.g. GP, to personal care by e.g. sports physician)

The secondary outcomes are:

- Subjective recovery after 1-year follow-up
- Pain at rest and during activity
- Function
- Return to sport
- Cost-effectiveness of the intervention
- Compliance of the intervention

### Measurements

During the one-year follow-up of the trAPP-study, measurements are scheduled monthly for 12 months after baseline. The participants will receive an e-mail that contains a secured hyperlink to the monthly questionnaire, using the survey application Lime Survey [19].

#### Baseline measurement

The baseline questionnaire includes questions on: demographics (age, gender, BMI, social economic status, co-morbidities), work activities (type, magnitude and load), daily and sport activities (Squash) [20], symptoms, function (AFS and FADI) [21,22], pain at rest and activity (11-point NRS) [23,24], medical care (treatment and diagnostics) and quality of life (EuroQol) [25].

#### Follow-up measurement

The follow-up questionnaires at 4, 8, 12, 26 and 52 weeks will include the following items: number of re-sprains, subjective recovery (measured on a 7-point Likert scale ranging from 1 ‘completely recovered’ to 7 ‘worse than ever’; patients are deemed to be recovered if they rate themselves as ‘fully recovered’ (=1) or ‘strongly recovered’ (=2) on the Likert scale, whereas those who rate themselves as ‘3, slightly recovered’ to ‘7, worse than ever’ are deemed to be not recovered), function (AFS and FADI) [21,22,26], pain at rest and activity (11-point NRS) [23,24], daily and sport activities (Squash) [20], quality of life (EuroQol) [25], treatment and diagnostics, direct and indirect costs. To assess direct costs patients are asked to report their health care consumption in the last 4 weeks. The health care consumption costs consist of a consultation with a physician, i.e. general practitioner, sports physician, physiotherapist, medical specialist (general surgeon or orthopedic surgeon), company doctor, remedial therapist or manual therapist. The number of consultations and the type of consultation (visit to a physician or therapist, contact by telephone, home visit or admission to the hospital) are monitored. In addition to the costs for consultation, the direct costs for the type of treatment provided by a physician or therapist and treatment applied by the patient himself are included (e.g. use of drugs, medical devices as tape, braces).

Indirect costs, defined as productivity costs, are the costs for absence from paid work and the costs for efficiency loss at unpaid work (study and household work), due to an acute ankle sprain. The total number of absent days from work and school is measured. Patients are asked to report the reason for their absence. By measuring the quality and quantity of the productivity level at paid work and/or school, the efficiency loss can be defined. Patients are asked, on a scale from 0 to 10, to report their productivity level of the quantity and quality of the work and school(work) done in the last 4 weeks. The total number of hours that housekeeping tasks were taken over by others and the number of hours paid help required due to an acute ankle sprain, are also reported.

For the control group, the follow-up questionnaire at 52 weeks, includes an extra question about possible use of the application ‘Versterk je Enkel’ during the one-year follow-up.

The follow-up questionnaires at 16, 21, 31, 35, 39, 43 and 47 weeks follow-up will only include the questions on re-sprains, recovery, function (AFS) and pain.

#### Compliance measurement

The compliance to the 8 weeks program in the intervention group will be measured by means of an online diary. The weekly follow-up diary measurements will gather information for each participant on the type of exercises performed and consequently the number, sets and repetitions of prescribed exercises undertaken. In addition questions will be asked about the clarity of the instructions and difficulty of the exercises. Subjects are defined to be compliant to the intervention when they have completed at least 75% of the training sessions [27].

### Analyses

All analyses will take place after the trial has finished, no intermediate analyses will be performed.

Descriptive statics will be applied to describe the patient characteristics, baseline values of the outcome measures of both groups and compliance of the intervention.

Differences between the intervention groups will be analysed following the intention-to-treat principle. Cox regression analysis will be used to compare ankle sprain recurrence risk between the intervention and control group. The secondary study parameters (pain scores, function) will be analyzed using regression techniques for repeated measures using generalized mixed models.

All analyses will be adjusted for baseline values and for co-interventions and possible prognostic factors in case the effect estimate changes with more than 10% when including these variables in the model.

#### Cost-analysis

The relevant costs are divided into direct costs (health care visits, medical devices, medication) and indirect costs (absenteeism from (un)paid work). A societal perspective is used for the economic evaluation.

The costs of drugs will be estimated on the basis of the prices recommended by the Royal Dutch Society of Pharmacy [28]. Other healthcare costs are valued using Dutch standard prices, based on the reference prices for direct costs within the healthcare sector [29].

Productivity costs will be measured by standard Dutch average productivity costs per hour, specified for sex and age [29].

Mean direct, indirect and total costs will be estimated and compared between the two groups, both for the costs per subject in the injured population and for the costs per subject in the total population. Because costs will not be normally distributed, 95% confidence intervals for the differences in mean costs will be obtained by bias corrected and accelerated bootstrapping (2000 replications). Differences in costs and differences in ankle sprain recurrences will be included in a cost-effectiveness ratio, which estimates the additional costs to prevent one ankle sprain recurrence. Confidence intervals for the cost-effectiveness ratio will be calculated with bootstrapping, using the bias-corrected percentile method with 5000 replications. An incremental cost-effectiveness ratio will be estimated of the incremental costs to prevent one ankle sprain recurrence. Uncertainty of this ratio will be evaluated by presenting a cost-effectiveness plane and sensitivity analyses will be performed to check the robustness of the results. An acceptability curve will also be presented.

## Discussion

The treatment of patients with an acute ankle sprain presenting in general practice is a challenge.

The Dutch guideline ‘Ligament injuries ankle’ for general practitioners contains potential treatments for acute ankle sprains. However, current clinical guidelines are not consistent and/or include non-evidence based treatments. An unsupervised neuromuscular training program for the prevention of re-sprains, studied among athletes, is possibly a useful and effective treatment tool for the GP [11]. In addition, the integration of this training program in an e-health supported program, can lead to an increase of the use of e-health supported preventive measures and interventions in usual care. The results of this study will therefore provide useful outcomes that can be used to revise the current Dutch and international guidelines, on the treatment of ankle sprains. If indeed, this e-health supported training program is effective in reducing the number of re-sprains in patients, a very relevant and easy to implement new intervention for patients with acute ankle sprains will become available for primary care.
